# Dermoscopy of Green Nail Syndrome: The “Green Aurora Sign”

**DOI:** 10.5826/dpc.1104a93

**Published:** 2021-10-01

**Authors:** Miguel Dominguez-Santas, Borja Diaz-Guimaraens, Juan Jimenez-Cauhe, Ana Suarez-Valle

**Affiliations:** 1 Dermatology Department, Ramon y Cajal University Hospital, Madrid, Spain

## Case Presentation

A 49-year-old woman presented to our dermatology department with a 1-year-long history of asymptomatic nail discoloration affecting the thumb of her right hand. She referred that she was treated with oral fluconazole by her general practitioner with no clinical improvement.

**Figure 1:**
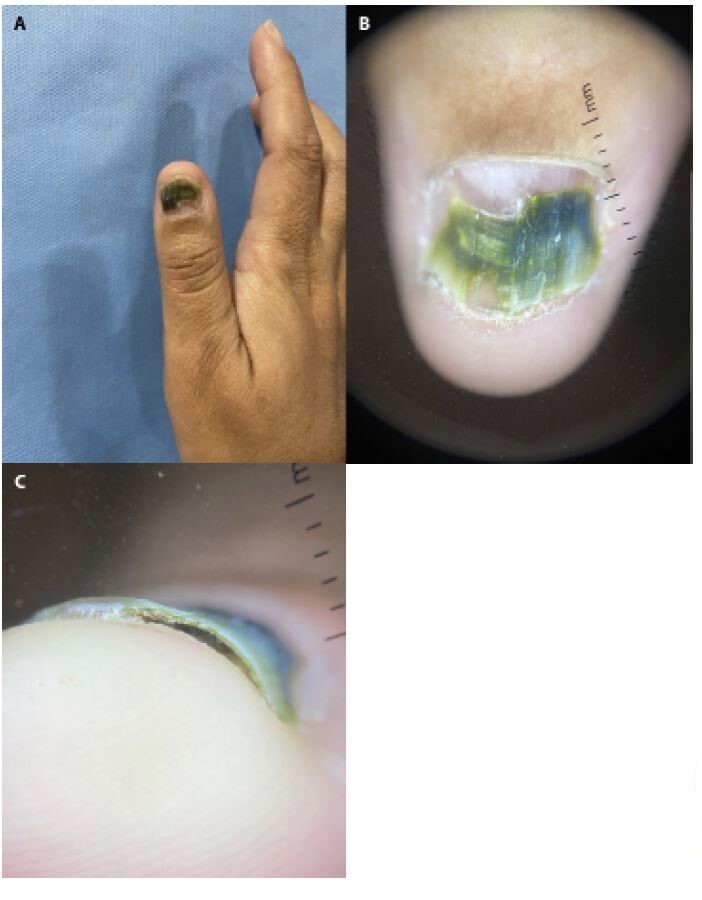
(A) Dark green discoloration of the nail plate. (B) Dermoscopy of the nail plate showed a brighter green discoloration with bluish hues. (C) Dermoscopy of the free edge showed distal onycholysis and the presence of the pigment in the ventral side of the plate.

Dermatological examination showed dark green discoloration of the nail plate (Figure 1A). Dermoscopy of the nail plate showed a brighter green discoloration with bluish hues (Figure 1B). Dermoscopy of the free edge showed distal onycholysis and the presence of the pigment in the ventral side of the plate (Figure 1C). The patient in this manuscript provided written informed consent to the publication of her case details.

## Teaching Point

Green nail syndrome is caused by the accumulation of pyocyanin that is produced by Pseudomonas aeruginosa bacterium [1]. Although it may be confused with onychomycosis, the absence of nail bed hyperkeratosis should guide the clinician towards the correct diagnosis. Onychomycosis can present the aurora borealis sign if dermoscopy is used [2], we therefore suggest using the term “Green aurora sign” to differentiate green nail syndrome dermoscopy from the one seen in onychomycosis.
